# M-Doped (M = Zn, Mn, Ni) Co-MOF-Derived Transition Metal Oxide Nanosheets on Carbon Fibers for Energy Storage Applications

**DOI:** 10.3390/nano14221846

**Published:** 2024-11-19

**Authors:** Andrés González-Banciella, David Martinez-Diaz, Adrián de Hita, María Sánchez, Alejandro Ureña

**Affiliations:** 1 Materials Science and Engineering Area, Escuela Superior de Ciencias Experimentales y Tecnología, Universidad Rey Juan Carlos, C/Tulipán s/n, 28933 Móstoles, Spain; 2 Instituto de Investigación de Tecnologías para la Sostenibilidad, Universidad Rey Juan Carlos, C/Tulipán s/n, 28933 Móstoles, Spain

**Keywords:** metal–organic frameworks, supercapacitors, Li-ion batteries, transition metal oxides, carbon fiber

## Abstract

Carbon fiber, with its strong mechanical properties and electrical conductivity, is ideal as a fiber electrode in wearable or structural energy storage devices. However, its energy storage capacity is limited, and coatings like transition metal oxides (TMOs) enhance its electrochemical performance. Metal–organic frameworks (MOFs) are commonly used to grow TMOs on carbon fibers, increasing the surface area for better energy storage. Despite this, TMOs have limited electrical conductivity, so ion exchange is often used to dope them with additional cations, improving both conductivity and energy storage capacity. This study compares different ion-exchange cations in ZIF-L-derived TMO coatings on carbon fiber. Testing both supercapacitor and Li-ion battery applications, Ni-doped samples showed superior results, attributed to their higher exchange ratio with cobalt. As a supercapacitor electrode, the Ni-doped material achieved 13.3 F/g at 50 mA/g—66% higher than undoped samples. For Li-ion battery anodes, it reached a specific capacity of 410.5 mAh/g at 25 mA/g, outperforming undoped samples by 21.4%.

## 1. Introduction

The development of all-solid-state energy storage devices has emerged as one of the most important challenges for the achievement of sustainable development goals [1]. The replacement of conventional flammable electrolytes with their new solid counterparts is not only a safety issue [2] but also an opportunity to achieve new functionalities [3,4,5]. All-solid-state batteries and supercapacitors could be wearable or even present mechanical properties, which allow their use not only as energy storage components but also as structural ones [6,7,8]. This multifunctionality could open the way for a new generation of structures with energy storage capability, which would be more efficient and sustainable [9]. Nevertheless, this goal is still far from being achieved due to the difficulty of finding materials that show a good relationship between electrochemical and mechanical performance [1,9]. In this context, carbon fiber (CF) fabrics have been proposed as a promising substrate for battery or supercapacitor electrodes due to their high elastic modulus, high mechanical resistance, excellent electrical conductivity, and low density [10,11]. So much so that even the bare carbon fiber fabric has been commonly used as an all-solid-state battery anode [12,13]. However, to improve the electrochemical performance of the device, the design of anodes that display higher capacities and capacitances is essential [10]. In fact, for this purpose, several coatings with other materials, which display higher energy storage capability, have been widely reported in the last years [14,15,16,17,18,19,20]. Transition metal oxides (TMOs) are known to be widely reported as battery anodes [21,22]. These materials are abundant, their cost is low, and, what is even more relevant, their specific capacity is about three times higher than that of conventional graphitic anodes [23]. In addition, TMOs are widely reported as supercapacitor (SC) electrode materials due to their chemical stability and larger capacitances due to pseudocapacitive reactions [24,25,26]. However, TMOs show two main disadvantages for these purposes. The first one is that TMOs present a relatively low electrical conductivity, which limits the electrode’s rate capability. Thus, bimetallic and bivalence TMOs have attracted more attention than their counterparts, which involve only a single-valence metal. So, the presence of several metals or metals in more than one oxidation state normally improves the electrical conductivity due to the activation energy for the electron transfer between cations being lower [27,28]. The second disadvantage is the poor cyclability of TMOs related to the volume expansion during the lithiation–delithiation process [21,23], which damages the material, worsening the electrical contact. In order to solve this problem, the use of porous TMOs has been demonstrated to be key. The synthesis of porous TMOs has been widely researched, with the obtention through metal–organic frameworks (MOFs) calcination a common way to achieve it [29,30,31]. MOFs exhibit large mesoporosity, which partially remains after the calcination [32]. Moreover, the porosity associated with the MOF-derived obtention improves the capacitance of TMOs as supercapacitor electrodes by the surface area [18]. Thus, this synthesis strategy has turned into an attractive way to improve TMO performance as both battery anodes [31,33] and supercapacitor electrodes [34]. In the last years, some examples of MOF-derived TMO coatings on carbon fiber have been reported [35,36,37,38,39,40]. The majority of works are based on cobalt and/or zinc zeolitic imidazole frameworks as the MOF precursor due to their demonstrated easy synthesis on carbon fiber and the excellent electrochemical performance of the derived Co_3_O_4_ and ZnCo_2_O_4_ spinels [35,36,37,38,39,40,41,42,43,44,45,46,47,48,49,50,51,52,53]. In this way, these materials have been demonstrated to be promising electrodes for all-solid-state Li-ion batteries [42,49,50,51,52,53] and supercapacitors [36,37,38,39,41,45,48,54]. Nevertheless, derived compounds obtained by the ion exchange of the MOF precursor with different cations have exhibited better performance [41,44,45,46]. In this work, Co-ZIF-L synthesized over carbon fiber fabric has been submitted to an ion exchange with Ni^2+^, Zn^2+,^ and Mn^2+^ cations in order to obtain M-doped Co_3_O_4_ (M = Ni, Mn, Zn) coatings and compare their electrochemical performance as both Li-ion battery anode and supercapacitor electrode for all-solid-state devices, studying the physicochemical reasons behind the differences.

## 2. Materials and Methods

### 2.1. Materials

Plain weaving (1 × 1) (193 g/m^2^) of 3K AS4 GP carbon fiber supplied by Hexcel^®^ (Stamford, EUUU). Nitric acid (HNO_3_) 65% *v*/*v* and zinc (II) nitrate hexahydrate (Zn(NO_3_)_2_·6H_2_O) ≥ 99% supplied by Sigma-Aldrich (Burlington, EEUU). 2-Methylimidazole (2-mIM) < 98% supplied by TCI (Tokyo, Japan). Cobalt (II) nitrate hexahydrate (Co(NO_3_)_2_·6H_2_O) 98.0–102.0%, Manganese (II) nitrate tetrahydrate (Mn(NO_3_)_2_·4H_2_O) 98%, and Nickel (II) nitrate hexahydrate (Ni(NO_3_)_2_·6H_2_O) 98% supplied by Thermo Fisher scientific (Waltham, EEUU).

### 2.2. TMO Synthesis on Carbon Fiber Fabric

First, ZIF-L MOF was synthesized on the carbon fiber fabrics to be used as a precursor for the later obtention of the TMO materials. For this purpose, CF fabrics of 4 × 4 cm were cut, and then the original coating of the fibers (commonly called sizing) was removed through immersion in acetone for 48 h. After that, CF fabrics were immersed in HNO_3_ for 1 h to generate polar groups on the surface through partial oxidation of the fibers [55]. Subsequently, fabrics were washed with distilled water and immersed in a Co(NO_3_)_2_ solution of 0.05 M for 10 min to favor the MOF growth on the CF surface through electrostatic interactions between the generated polar groups and Co^2+^ cations [42,56]. Having passed this time, CF pieces were removed from the solution, and 2-methylimidazole (0.4 M) was added to the previous solution. Finally, after mixing both solutions for 1 min, the carbon fiber fabrics were introduced into the mixture for 1 h, washed with distilled water, and dried overnight at 60 °C. Moreover, the synthesized MOF powder particles, which did not adhere to the CF or did not begin to grow on it, were filtered, washed, and dried using the same method as the CF fabrics. The next step involved ion exchange. CF samples were individually introduced into 0.01 M solutions of M(NO_3_)_2_ (M = Zn, Mn, and Ni) in ethanol for 10 min. After drying, all the samples were submitted to a heat treatment to obtain the corresponding TMOs from the MOF coatings. To evaluate the effect on the final performance of the three proposed different ion-exchange steps, samples without ion exchange were also subjected to the same thermal treatment. More in detail, the heat treatment consisted of two steps. The first one was annealing at 500 °C for 1 h in the Ar atmosphere to generate oxygen vacancies, which improve the electrical conductivity [40]. The second one involved a calcination step at 350 °C for 30 min with the aim of transforming the MOF material into the final TMO coating. The samples were named Zn-doped TMO, Mn-doped TMO, Ni-doped TMO, and undoped TMO. As references, powders collected in the synthesis underwent the same ion exchange in ethanol solutions with M(NO_3_)_2_ (M = Zn, Mn, and Ni), followed by the same heat treatments.

### 2.3. Material Characterization

Scanning electron microscopy (SEM) images from MOF precursor coatings on carbon fiber were taken by S-3400 N by Hitachi (Chiyoda, Japan), while SEM images of TMO coatings on the carbon fiber were taken using a PRISMA-E by Thermo Fisher (Waltham, EEUU). Transmission electron microscopy (TEM) images were acquired using STEM F200 by JEOL (Tokyo, Japan). X-ray diffraction (XRD) patterns were acquired from the reference powders by X’PERT diffractometer by PHILIPS (Amsterdam, Netherlands), using Cu Kα radiation. Moreover, X-ray photoelectronic spectra (XPS) were collected by VersaProbe II by PHI (Chanhassen, EEUU) using Al 1486.6 eV mono at 47.3 W as an X-ray source.

### 2.4. Supercapacitor Electrode Characterization

The electrochemical performance of the samples as a supercapacitor electrode was evaluated in a three-electrode system in which the counter-electrode was a Pt electrode, the reference electrode an Ag/AgCl electrode, and the electrolyte was a KOH 2 M solution. Cyclic voltammetry (CV) and galvanostatic charge–discharge (GCD) measurements were acquired with an Autolab PGSTAT302N potentiostat, being the potential window between 0 and 0.45 V. The studied current densities were 50, 100, 150, 200 and 250 mA/g. Additionally, the stability of the electrodes was tested by measuring the specific capacitance after 5000 GCD cycles at 150 mA/g of current density using a NEWARE battery testing system BTS4000-5V10M. The specific capacitance values ($C_{s}$) were calculated from CV as:

(1) $$C_{s}=\frac{\int_{V_{min}}^{V_{max}}idV}{2\;\nu \;V_{0}},$$

where $V_{0}$ is the potential window, ν the scan rate, and i the current density. On the other hand, the specific capacitance was also calculated from GCD results as:

(2) $$C_{s}=\frac{it}{V_{0}},$$

where t is the time of the discharge. The masses of the electrodes were 0.0021 g for the Undoped sample, 0.0019 g for the Zn-doped sample, 0.0019 g for the Mn-doped sample, and 0.0019 g for the Ni-doped one.

### 2.5. Li-Ion Battery Anode Characterization

Electrochemical measurements of TMO coatings on CF for Li-ion anodes were performed in half-cell configuration coins 2025. Lithium metal served as counter-electrode, and glass microfiber filters with a diameter of 150 mm, supplied by Whatman, were used as separators. Coins were assembled into an Ar-filled glovebox to avoid O_2_ and H_2_O presence. A 1 M LiPF_6_ electrolyte in ethylene carbonate and dimethyl carbonate (1:1 *v*/*v*) supplied by Merck was used. Galvanostatic charge–discharge (GCD) tests were performed using a NEWARE battery testing system BTS4000-5V10MA. To determine the specific capacity and evaluate the rate capability and capacity retention, tests consisting of five cycles at different current densities were performed (25, 50, 100, 250, 500, and 25 mA/g). Additionally, to fully evaluate the capacity retention, 100 GCD cycle tests were carried out at 100 mA/g to evaluate the long-term performance of the batteries. Additionally, cyclic voltammetry (CV) and electrochemical impedance spectroscopy (EIS) tests were conducted using an Autolab PGSTAT302N potentiostat. The potential window of CV tests was from 0 to 3 V, while EIS measures were taken at 0.01 V between 0.1 and 105 Hz.

In this case, the specific capacity values were calculated from GCD results as follows:

(3) $$C_{s}=it$$

The masses of the electrodes were 0.0022 g for the undoped sample, 0.0019 g for the Zn-doped sample, 0.0020 g for the Mn-doped sample, and 0.0017 g for the Ni-doped one.

## 3. Results

### 3.1. Coating Characterization

**Figure 1:**
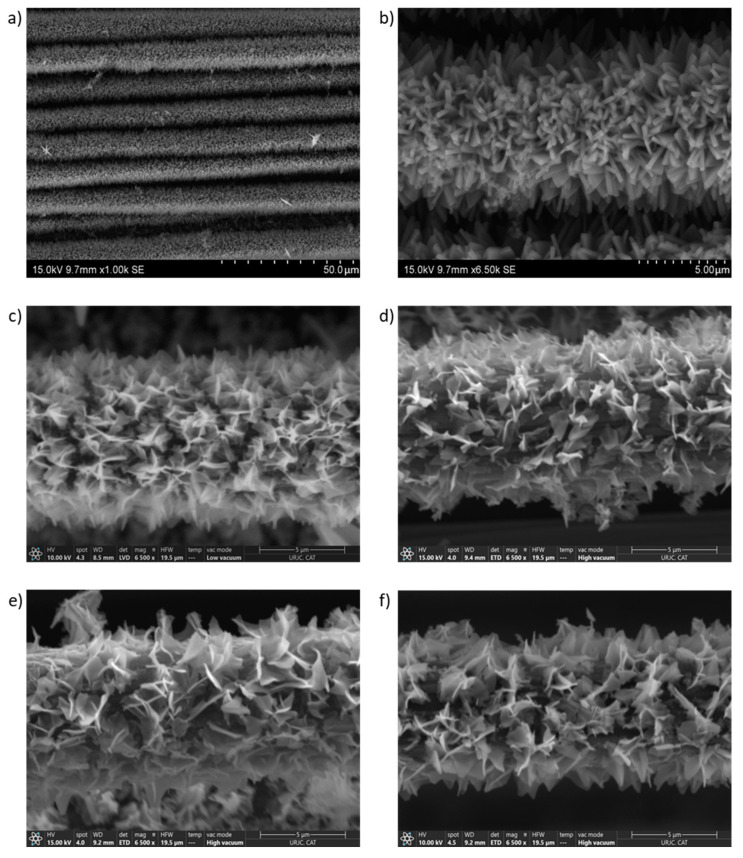
SEM images of ZIF-L coating on carbon fiber fabric at (**a**) lower magnification and (**b**) higher magnification, (**c**) undoped TMO sample, (**d**) Zn-doped TMO sample, (**e**) Mn-doped TMO sample, and (**f**) Ni-doped TMO sample.

In Figure 1, the obtained coating morphology was examined by SEM. Figure 1a shows a general image of the ZIF-L MOF coating on the CFs, demonstrating both its homogeneity and the high density achieved. As previously detailed, this MOF coating will be used as a precursor for the later formation of the different TMO materials. Furthermore, it can be observed how the MOF coats each CF instead of being a continuous layer over the tows. This kind of coating could be advantageous in ensuring a good interface between the CF fabric electrode and the solid polymer electrolyte. Figure 1b shows a more detailed image of the ZIF-L coating, revealing triangular nanosheets with nanometric thickness, around 1 µm in size. The resulting TMO without ion exchange (undoped TMO) after the heat treatment is shown in Figure 1c, where some similarities and differences in comparison with the ZIF-L precursor coating can be analyzed. The coating retains its high density, homogeneity, and nanosheet morphology, which provide a high surface area that is essential for good electrochemical performance, especially for supercapacitor applications. Nevertheless, the thickness is lower, and the nanosheets appear less defined after the heat treatment. Finally, Figure 1d–f show the resulting TMO coating with the different proposed ion exchanges: Zn-doped, Mn-doped, and Ni-doped, respectively. As can be observed, no significant differences in the morphology of the coating were detected between the undoped and doped TMO coatings.

**Figure 2:**
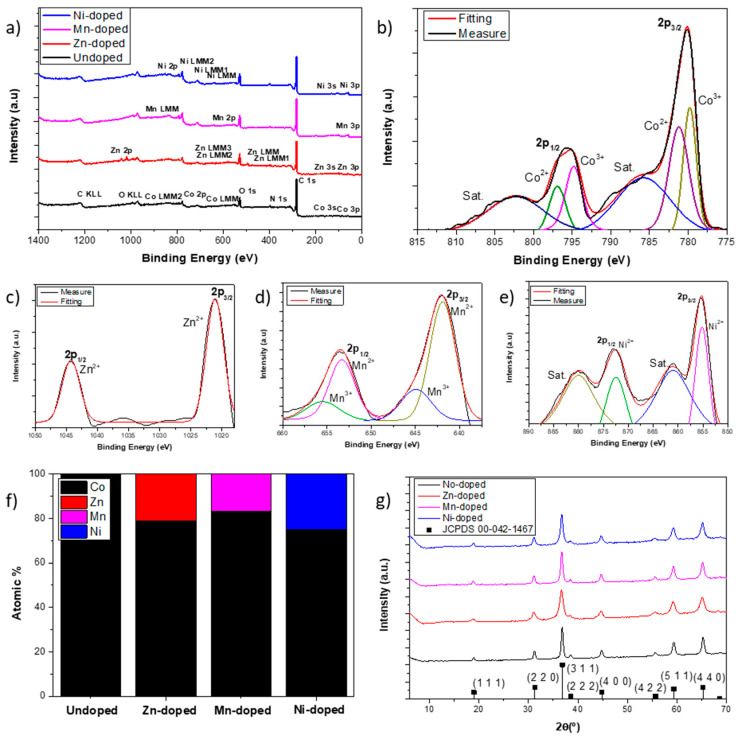
(**a**) XPS surveys. (**b**) XPS Co 2p core level of the undoped sample. (**c**) XPS Zn 2p core level of Zn-doped sample. (**d**) XPS Mn 2p core level of Mn-doped sample. (**e**) XPS Ni 2p core level of Ni-doped sample. (**f**) The atomic percentage of each cation in each sample. (**g**) XRD spectra of TMO powder samples.

In order to make sure that ion exchange has occurred satisfactorily and to analyze the composition of each sample, XPS and EDS spectra were collected and presented in Figure 2a and Figure S1, respectively. Both EDS spectra and XPS surveys demonstrated the presence of the dopant cations (Ni^2+^, Zn^2+^, and Mn^2+^) in the corresponding samples. Figure 2b shows the Co 2p core level of the undoped sample. Two main peaks, located about 790 and 795.6 eV, correspond to 2p_3/2_ and 2p_1/2_, respectively, while the wider peaks, located at 786 and 802.3 eV, are the corresponding satellites of these levels. However, the main peaks can be deconvoluted into the other two peaks, each one obtaining four peaks at 779.8, 794.8, 781.2, and 797.0 eV. The first two peaks are attributed to Co^3+^, and the other two are associated with Co^2+^ [36,57]. The presence of the two oxidation states suggests that the composition of the undoped TMO could be Co_3_O_4_. Figure 2c–e show the Zn 2p, Mn 2p, and Ni 2p core levels of the ion-exchanged samples Zn-doped, Mn-doped, and Ni-doped, respectively. In these core levels, it is realizable that all dopant cations show a fully or very majority 2+ oxidation state. The cation atomic contribution was quantified, and the results are summarized in Figure 2f. Ni exchanged to Co by 25.3%, being the highest among the dopants, possibly due to its size similarity to Co, which better suits its positions than the others. Moreover, Zn exchanged to Co by 21.1% while Mn only by 16.9% due to its larger size, which makes it difficult not only to exchange in the crystalline structure but also the intercalation. Having demonstrated that the ion exchange occurs for all dopant cations, XRD analysis was performed and summarized in Figure 2g to determine the crystalline structure and how it is affected by the ion exchange. In regard to undoped TMO, the XRD spectra match the expected XRD for Co_3_O_4_ spinel, while the doped-TMOs present slight differences with respect to the undoped one. Thus, XRD spectra indicate the same crystalline structure for all obtained TMOs. However, some conclusions can be drawn from them. Taking into account the fact that, according to XPS analysis, the doping in neither case reaches 33% of total cations and XRD results, the obtained TMOs are Co_3_O_4_ for the undoped sample, ZnCo_2_O_4_@Co_3_O_4_ for the Zn-doped sample, MnCo_2_O_4_@Co_3_O_4_ for the Mn-doped sample, and NiCo_2_O_4_@Co_3_O_4_ for the Ni-doped one. Dopant cations distort spinel unit cells, so crystallinity is affected by the ion exchange. As a result of the great difference in size between Co^2+^ and Zn^2+^, the Zn-doped sample presents wider peaks, which indicate lower crystallinity. Nevertheless, in spite of being Ni^2+^, the more similar cation to Co^2+^ in size, the Mn-doped sample presents more crystallinity due to the low exchange degree.

**Figure 3:**
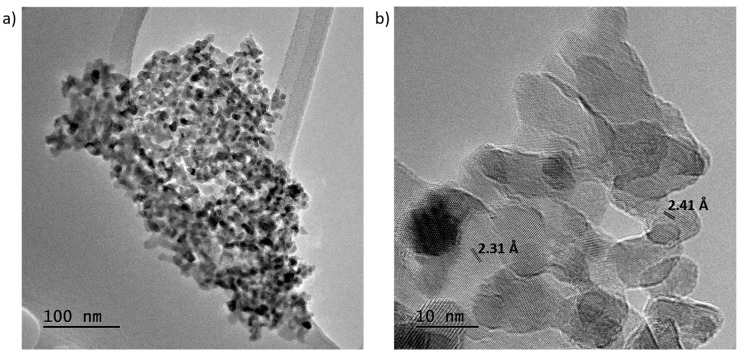
TEM images of undoped TMO samples at (**a**) lower and (**b**) higher magnification.

Additionally, in order to study the microstructure, TEM images of the undoped TMO are presented in Figure 3. It is possible to observe that the nanosheets are composed of little nanocrystals with a diameter of around 10 nm, displaying high crystallinity. Although diffraction patterns were not possible to obtain due to the small grain size, some interplanar distances were measured in real space. One of them was 2.31 Å and the other 2.41 Å. Both could correspond to Co_3_O_4_ spinel structure, the first one to the plane (2 2 2) and the second one to the plane (3 1 1), confirming XRD crystal structure results.

### 3.2. Characterization as Supercapacitor Electrode

**Figure 4:**
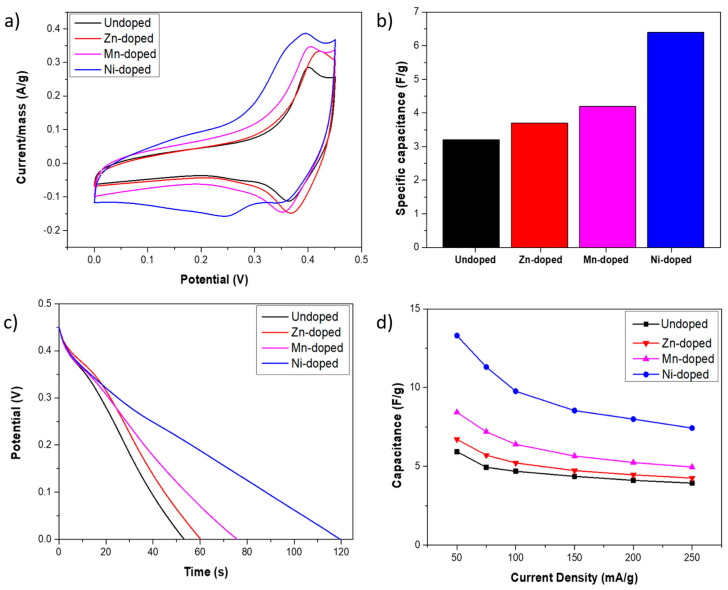
(**a**) CV curves at a scan rate of 20 mV/s. (**b**) Specific capacitance values are calculated from CV at 20 mV/s. (**c**) GCD test discharges at 50 mA/g. (**d**) Specific capacitance calculated from GCD tests at different current densities.

After compositional and morphological characterization of the synthesized TMO coatings, CV tests were performed to compare the electrochemical processes of the samples acting as supercapacitor electrodes. Figure 4a shows a comparison of CV curves for all samples at a scan rate of 20 mV/s, revealing that all samples displayed an oxidation peak at 0.4 V and another reduction peak at 0.36 V, indicating a pseudocapacitance energy storage mechanism. These peaks can be associated with the reversible faradaic reactions (4) and (5) [58,59]:

*MCo*_2_*O*_4_ + *H*_2_*O* + *OH*^−^ ↔ *MOOH* + 2*CoOOH* + *e*^–^ (4)

*CoOOH* + *OH*^−^ ↔ *CoO*_2_ + *H*_2_*O* + *e*^−^ (5)

The material that exhibited the lower current at these peaks was the undoped one due to the presence of other cations on cobaltite spinels, resulting in a lattice distortion that improves the electrochemical activity [60], being the undoped material the only one that does not exhibit this effect. Moreover, in the Ni-doped sample, the oxidation peak appears to consist of two different peaks, one at 3.95 V and the other at 3.6 V, while another reduction peak at 0.24 V, hardly visible on the other sample curve, is evident. These peaks are related to the reaction (6) [61,62,63].

*NiOOH* + *OH*^−^ ↔ *NiO* + *H*_2_*O* + *e*^–^ (6)

Figure 4b summarizes the calculated specific capacitance displayed for each sample from CV curves at 20 mV/s, considering the mass as the whole mass of the electrode. Because of the aforementioned additional reactions, the Ni-doped sample displayed a larger specific capacitance, achieving 6.4 F/g per gram of the whole electrode. Nevertheless, all doped samples exhibited larger specific capacitance than the undoped one, which only displayed 3.2 F/g, while the Zn-doped and Mn-doped displayed 3.7 F/g and 4.6 F/g, respectively. In order to evaluate the electrochemical performance of the samples as supercapacitor electrodes, GCD tests were carried out and represented in Figure S2. Figure 4c depicts only discharges at 50 mA/g, facilitating the comparison. The above-discussed reduction peak at 0.36 V is observable as a plateau in the GCD discharge in all cases. Nonetheless, the plateau at 0.24 V is also observable in the Ni-doped sample curve, as was expected from CV results. In addition, it is possible to obtain information about the internal resistance from GCD discharge curves that are associated with the IR drop. Analyzing the discharge curve of the CGD test (Figure S3), performed from 50 to 250 mA/g, it can be concluded that the IR drops at 250 mA/g are more evident. These IR drops and their associated internal resistance are summarized in Table 1. The results suggest that the Zn-doped sample displays the lower electrical resistance, followed by the Ni-doped, Mn-doped, and undoped ones, respectively. Moreover, the specific capacitances were also evaluated by GCD tests, and the results are summarized in Figure 4d. Inconsistent with CV tests, Ni-doped samples exhibited the highest capacitance values at all studied current densities. For instance, at a current density of 50 mA/g, it reached 13.3 F/g, while the Mn-doped, Zn-doped, and undoped samples achieved 8.4, 6.7, and 5.9 F/g, respectively. This represents a 37%, 50%, and 66% decrease compared to the Ni-doped sample. Even at a current density of 250 mA/g, the Ni-doped sample maintained a specific capacitance of 7.4 F/g, which is 56% of the value observed at 50 mA/g. Thus, Ni-doped sample electrochemical performance as a supercapacitor electrode was further characterized due to its superior performance in this application.

**Figure 5:**
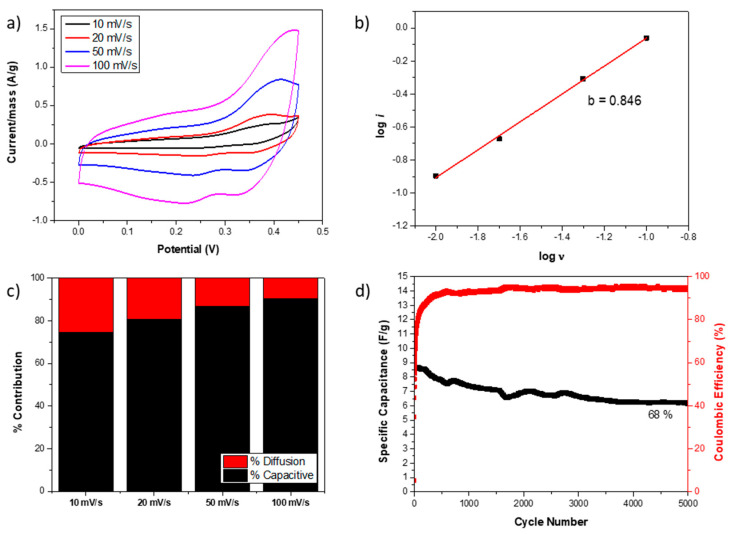
(**a**) CV curve of Ni-doped sample at different scan rates and (**b**) calculated *b* value in the inset. (**c**) Diffusion and capacitive-controlled processes contribute to different scan rates for Ni-doped samples. (**d**) Specific capacitance and coulombic efficiency of Ni-doped sample after 5000 GCD cycles at 150 mA/g.

Kinetic information about the electrode can be obtained from CV tests. The current of a peak (*i*) increases proportionally to the scan rate (*ν*) when the process is totally capacitive-controlled, while it increases proportionally to the square root of the scan rate when the process is totally diffusion-controlled. Then, it is possible to understand the dependence of the current peak with the scan rate as detailed in Equation (7):

*i* = *av*
^*b*^ (7)

where *a* and *b* are coefficients. The *b* coefficient is between 0.5 and 1; a *b* value close to 0.5 indicates a more diffusion-controlled process, while a *b* close to 1 is a more capacitive-controlled process [64]. Figure 5a represents the CV curves of the Ni-doped sample at different scan rates, and Figure 5b illustrates the *log*(*i*) versus *log*(*ν*^1/2^), whose slope is the coefficient *b*. The obtained *b* value was 0.85, suggesting a more capacitive-controlled process. Moreover, the current peak can be understood as the sum of two contributions [30] following Equation (2): one related to capacitive-controlled processes, which depends on the scan rate (*ν*), and the other to diffusion-controlled processes, which depends on the square root of the scan rate (*ν*^1/2^).

*i* = *i_cap_* + *i_dif_* = *k*_1_*v* + *k*_2_*v*
^1/2^ (8)

where the parameters *k*_1_ and *k*_2_ can be calculated by the lineal regression obtained from the *i*/*ν* ^1/2^ versus the *ν* ^1/2^ in order to calculate the contribution of each process at different scan rates, as it is represented in Figure 5c for the Ni-doped electrode. As was expected because of the calculated *b* value, the behavior is mainly controlled by capacitive processes rather than diffusion processes. However, as the scan rate rises, the contribution of capacitive-controlled processes increases further, primarily due to the kinetic inhibition of diffusion at high scan rates. On the other hand, the Ni-doped specific capacitance and the coulombic efficiency along 5000 GCD cycles at 150 mA/g are shown in Figure 5d to evaluate the capacitance retention. The reached coulombic efficiency was 94%, indicating the redox process was partially irreversible. Because of that, the initial specific capacitance, which was 9 F/g, was reduced by 32% after 5000 GCD cycles. Nevertheless, this value appears to stabilize, and it is still higher than the specific capacitances displayed by the undoped, Zn-doped, and Mn-doped samples in only one GCD test.

### 3.3. Characterization as Li-Ion Battery Anode

**Figure 6:**
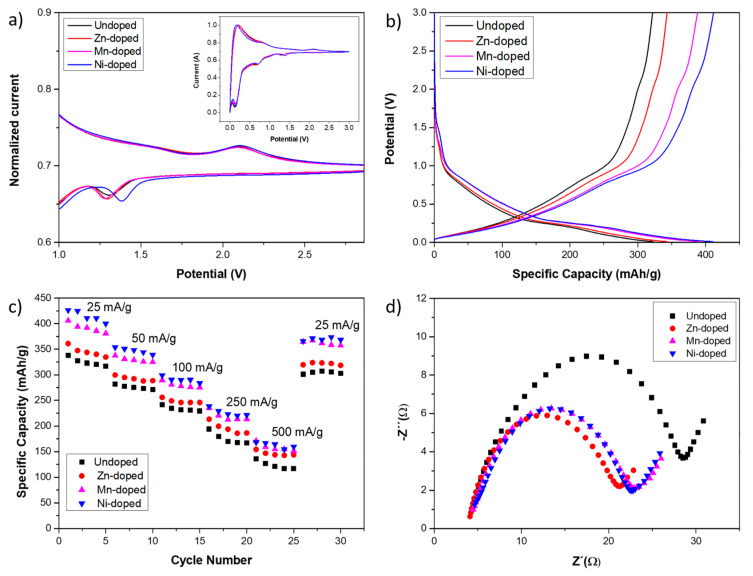
(**a**) Normalized third-cycle CV of the samples. (**b**) GCD curves at 25 mA/g of the samples. (**c**) GCD calculated capacities at different current densities. (**d**) Nyquist plots of the samples.

After evaluating the behavior of the different TMOs as an electrode for supercapacitors, their performance as an anode in Li-ion batteries was also investigated to explore the versatility of the synthesized TMO materials for diverse energy storage devices. In order to study the TMO coating electrochemical reactions, CV tests were carried out. Figure 6a shows the current normalized CV curves, enabling a comparative analysis of the coating peaks. Peaks associated with MCo_2_O_4_@Co_3_O_4_ coatings are those located around 1.3 V in the cathodic process and around 1.6 and 2.2 V in the anodic process, which are being expanded in the figure, while the others, presented in the inset, can be attributed to the carbon fiber [44,51,52,53]. In the cathodic process, the peak around 1 V is related to the reduction in MCo_2_O_4_ and Co_3_O_4_ to metallic M and Co. In the anodic process, the peak at around 1.6 V is related to the oxidation of M and Co to M^2+^ and Co^2+^, and the peak around 2.2 V to the oxidation of Co^2+^ to Co^3+^. Thus, the involved electrochemical reactions during the charge–discharge process are the following:

*MCo*_2_*O*_4_ + 8*Li*^+^ → *M* + 2*Co* + 8*Li*_2_O (9)

*Co*_3_*O*_4_ + 8*Li*^+^ → 3*Co* + 4*Li*_2_*O* (10)

*M* + *Li*_2_*O* → *MO* + 2*Li*^+^ (11)

*Co* + *Li*_2_*O* → *CoO* + 2*Li*^+^ (12)

2*CoO* + *MO* + *Li*_2_*O* → *MCo*_2_*O*_4_ + 2*Li*^+^ (13)

3*CoO* + *Li*_2_*O* → *Co*_3_*O*_4_ + 2*Li*^+^ (14)

The main difference among CV curves of the diverse samples is related to the cathodic peak located around 1.3 V. This peak is narrower in CVs obtained from doped samples in comparison to undoped ones, indicating a better electrical conductivity induced by the cation substitution. These CV curves can be correlated to GCD curves, where each electrochemical reaction, manifested as a peak in CV, is represented in the GCD curve as a plateau. Figure 6b shows the GCD curves at a current density of 25 mA/g collected from the samples. The anodic reaction at 1.6 V is hardly observable as a change in the curvature during the charge process, while the reaction at 2.2 V is evident. The cathodic reaction at 1.3 V is observable during the discharge process. Figure 6c shows the calculated capacities, where it can be observed how the Ni-doped sample displayed the highest capacity value across all cases; for instance, at a current density of 25 mA/g, it reached 410.5 mAh/g, whereas the Mn-doped sample exhibited 392 mAh/g, Zn-doped 346.3 mAh/g, and undoped 322.7 mAh/g. These values are lower by 4.5%, 15.7%, and 21.4% compared to the Ni-doped sample, respectively. Assessing the rate capability, i.e., the retention of the capacity as the current density increases, the undoped sample retains 37.6% of its capacity, while the Zn-doped one retains 41.8%, the Mn-doped retains 39.4%, and the Ni-doped retains 40%, as the current density increases from 25 to 500 mA/g. This parameter is related to the electrical conductivity, suggesting that the most electrically conductive sample is Zn-doped, followed by Ni-doped and Mn-doped, whereas the undoped sample, being the monometallic TMO, exhibits the least conductivity. Nevertheless, it is important to realize that even at a current density of 500 mA/g, the Ni-doped sample capacity is higher than Mn-doped, Zn-doped, and undoped samples by 5.9%, 12.5%, and 26.1%, respectively. Moreover, the capacity retention can also be evaluated from the results of Figure 6c, relating the initial capacity at 25 mA/g with the capacity at the same current density after the samples have been tested at the other current densities. The undoped sample retains the most capacity, 95.1%, suggesting that the doping with other cations decreases the cyclability, which could be a consequence of the distortions in the crystalline structure. However, smaller cations like Zn^2+^ decrease cyclability by only 6% compared to larger ones like Mn^2+^, which, despite being less exchanged, lose only 7.7% of the initial capacity. Owing to the large degree of exchange, the Ni-doped sample exhibits the least capacity retention, losing 9.3%. Nevertheless, again, it is important to realize that despite these results, even after all these tests, the Ni-doped sample still demonstrates the highest specific capacity.

Finally, EIS tests were carried out to evaluate the charge transfer resistance. The Nyquist plots of each sample are presented in Figure 6d, while the results are summarized in Table 2. Typically, the charge transfer process between electrode and electrolyte is related to the first semicircle [35], and its resistance can be calculated as its width. The charge transfer resistance value was 28.5 Ω for the undoped sample, 19.4 Ω for the Zn-doped sample, 20.6 Ω for the Mn-doped sample, and 20.2 Ω for the Ni-doped sample. These results agree with the rate capability discussion presented above, wherein lower electrical resistance was expected for the Zn-doped sample and higher resistance for the undoped one, while Ni-doped and Mn-doped are expected to present similar values.

**Figure 7:**
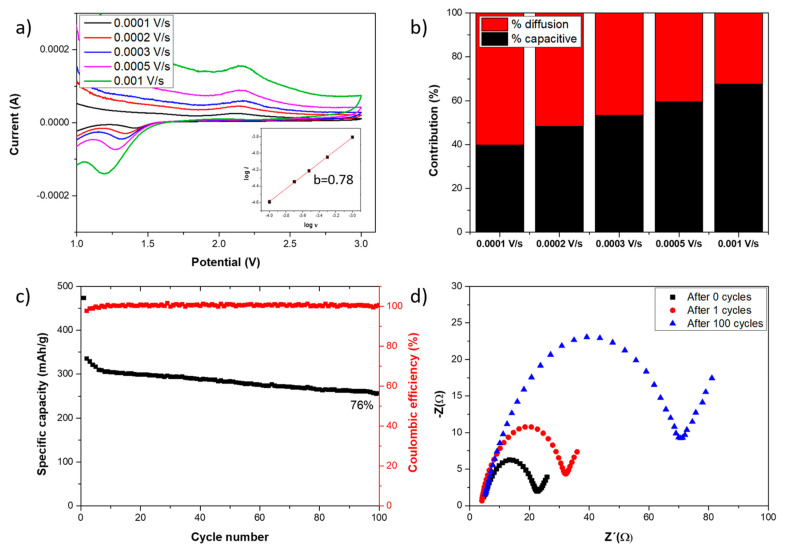
(**a**) Ni-doped sample CV at different scan rates and calculated b value in the inset. (**b**) Percentage of each contribution at different scan rates in the Ni-doped sample. (**c**) Specific capacity of Ni-doped sample after 100 GCD cycles at 100 mA/g. (**d**) Nyquist plot of Ni-doped sample after several number of cycles.

Owing to its better performance as a Li-ion battery anode, the Ni-doped sample was more extensively characterized. Figure 7a shows Ni-doped sample CV at different scan rates, focusing on NiCo_2_O_4_@Co_3_O_4_ characteristic peaks and the b value in the inset, which was 0.78. This *b* value indicates a half behavior between capacitive and diffusion-controlled processes. In Figure 7b, capacitive and diffusion-controlled contributions are represented for all tested scan rates. It is observable how the capacitive contribution is higher as the scan rate increases due to high scan rates kinetically preventing diffusion. On the other hand, the cyclability of the Ni-doped electrode was evaluated through GCD 100 cycles at 100 mA/g of current density, being the specific capacity evolution shown in Figure 7c, just as the coulombic efficiency. The prominent decrease after the first cycle, where capacity is reduced by 29%, is attributed to the formation of the solid electrolyte interphase (SEI) layer. Following the next five cycles, the capacity rapidly decreases by another 8%. After the 6th cycle, the decrease is continuous until reaching 76% of the value at the second cycle by the 100th cycle. Additionally, the coulombic efficiency was about 100% at all cycles except at the first, so the SEI formation is an irreversible process. EIS tests were also carried out to study the evolution of the charge transfer resistance as the cycle number increased. Figure 7d shows the Nyquist plot of the fresh sample after the first cycle and after 100 cycles; the result is summarized in Table 3. Attending to the charge transfer resistances, measured values were 20.2 Ω, 32.4 Ω, and 74.8 Ω, respectively. The first increment of the resistance is due to the SEI formation, while the increment after that is related to the degradation of the TMO during Li intercalation–deintercalation processes [65].

## 4. Conclusions

In this work, homogeneous and dense coatings with different TMOs have been developed on the carbon fiber through a Co-ZIF-L MOF precursor and ion exchange with Zn^2+^, Mn^2+,^ and Ni^2+^ as dopant cations. All doped samples have improved undoped TMO coating electrochemical performance, both as supercapacitor electrodes and Li-ion battery anodes. This enhancement can be attributed to the increased electrical conductivity caused by the bimetallic composition and the improved electrochemical activity as a consequence of the lattice distortion. As a supercapacitor electrode, the Ni-doped sample exhibited extra peaks with respect to other samples, attributed to the redox couple NiOOH/NiO, which is possibly due to its higher exchange degree. These extra peaks provided a Ni-doped sample with higher specific capacitance than its counterparts, 13.3 F/g of the electrode in the GCD test at 50 mA/g. This value was 37%, 50%, and 66% higher than Mn-doped, Zn-doped, and undoped samples, respectively. On the other hand, Ni-doped electrochemical performance was also higher than their counterparts as Li-ion battery anodes. The specific capacity of this sample was 410.5 mAh/g at a current density of 25 mA/g, which means 4.5%, 15.7%, and 21.4% more than Mn-doped, Zn-doped, and undoped samples, respectively. Thus, the results of this work highlight Ni^2+^ as the most suitable dopant cation for achieving enhanced electrochemical performance in both applications, emerging this Ni-doped cobaltite ZIF-L-derived as a promising coating on the carbon fiber fabrics to develop supercapacitor electrodes and Li-ion battery anodes for structural energy storage devices.

## Figures and Tables

**Table 1:** IR drops at a current density of 250 mA/g.

	Undoped	Zn-Doped	Mn-Doped	Ni-Doped
**IR drop**	67 mV	55 mV	64 mV	58 mV
**Internal** **Resistance**	0.134 Ω	0.110 Ω	0.128 Ω	0.116 Ω

**Table 2:** Summary of the EIS calculated resistances from different samples.

	Undoped	Zn-Doped	Mn-Doped	Ni-Doped
**Charge-transfer ** **Resistance (R_ct_)**	28.5 Ω	19.4 Ω	20.6 Ω	20.2 Ω
**Serial Resistance (R_s_)**	4.0 Ω	3.9 Ω	3.8 Ω	3.6 Ω

**Table 3:** Summary of the EIS calculated resistances of the Ni-doped sample after different number of cycles.

	Fresh	After the First Cycle	After 100 Cycles
**Charge-transfer resistance (R_ct_)**	20.2 Ω	32.4 Ω	74.8 Ω
**Serial resistance (R_s_)**	3.6 Ω	4.0 Ω	3.7 Ω

## Data Availability

Data will be made available on request.

## Supplementary Materials

The following supporting information can be downloaded at: https://www.mdpi.com/article/10.3390/nano14221846/s1, Figure S1: EDS spectra of (a) no-doped sample, (b) Zn-doped sample, (c) Mn-doped sample, and (d) Ni-doped sample; Figure S2: GCD test at different current densities of (a) undoped sample, (b) Zn-doped sample, (c) Mn-doped sample, and (d) Ni-doped sample; Figure S3: Galvanostatic discharges at different current densities of (a) undoped sample, (b) Zn-doped sample, (c) Mn-doped sample, and (d) Ni-doped sample.

## References

[1] Danzi F., Salgado R.M., Oliveira J.E., Arteiro A., Camanho P.P., Braga M.H. (2021). Structural batteries: A review. Molecules.

[2] Kalnaus S., Asp L.E., Li J., Veith G.M., Nanda J., Daniel C., Chen X.C., Westover A., Dudney N.J. (2021). Multifunctional approaches for safe structural batteries. J. Energy Storage.

[3] Kalita G., Endo T., Nishi T. (2023). Recent development on low temperature synthesis of cubic-phase LLZO electrolyte particles for application in all-solid-state batteries. J. Alloy Compd..

[4] Tuo K., Sun C., Liu S. (2023). Recent Progress in and Perspectives on Emerging Halide Superionic Conductors for All-Solid-State Batteries. Electrochem. Energy Rev..

[5] Yang X., Yin Q., Wang C., Doyle-Davis K., Sun X., Li X. (2023). Towards practically accessible high-voltage solid-state lithium batteries: From fundamental understanding to engineering design. Prog. Mater. Sci..

[6] Snyder J., Gienger E., Wetzel E. (2015). Performance metrics for structural composites with electrochemical multifunctionality. J. Compos. Mater..

[7] González C., Vilatela J., Molina-Aldareguía J., Lopes C., Llorca J. (2017). Structural composites for multifunctional applications: Current challenges and future trends. Prog. Mater. Sci..

[8] Greenhalgh E.S., Nguyen S., Valkova M., Shirshova N., Shaffer M.S., Kucernak A. (2023). A critical review of structural supercapacitors and outlook on future research challenges. Compos. Sci. Technol..

[9] Jin T., Singer G., Liang K., Yang Y. (2023). Structural batteries: Advances, challenges and perspectives. Mater. Today.

[10] Zhang S., Xiao S., Li D., Liao J., Ji F., Liu H., Ci L. (2022). Commercial carbon cloth: An emerging substrate for practical lithium metal batteries. Energy Storage Mater..

[11] Gulzar U., Goriparti S., Miele E., Li T., Maidecchi G., Toma A., De Angelis F., Capiglia C., Zaccaria R.P. (2016). Next-generation textiles: From embedded supercapacitors to lithium ion batteries. J. Mater. Chem. A.

[12] Asp L.E., Bouton K., Carlstedt D., Duan S., Harnden R., Johannisson W., Johansen M., Johansson M.K.G., Lindbergh G., Liu F. (2021). A Structural Battery and its Multifunctional Performance. Adv. Energy Sustain. Res..

[13] Moyer K., Meng C., Marshall B., Assal O., Eaves J., Perez D., Karkkainen R., Roberson L., Pint C.L. (2020). Carbon fiber reinforced structural lithium-ion battery composite: Multifunctional power integration for CubeSats. Energy Storage Mater..

[14] Yao S., Zhang G., Zhang X., Shi Z. (2020). Mace-like carbon fibers@Fe_3_O_4_@carbon composites as anode materials for lithium-ion batteries. Ionics.

[15] Huang Y., Yang H., Xiong T., Adekoya D., Qiu W., Wang Z., Zhang S., Balogun M.-S. (2020). Adsorption energy engineering of nickel oxide hybrid nanosheets for high areal capacity flexible lithium-ion batteries. Energy Storage Mater..

[16] Han Q., Zhang W., Han Z., Wang F., Geng D., Li X., Li Y., Zhang X. (2019). Preparation of PAN-based carbon fiber@MnO2 composite as an anode material for structural lithium-ion batteries. J. Mater. Sci..

[17] Subhani K., Hameed N., Al-Qatatsheh A., Ince J., Mahon P.J., Lau A., Salim N.V. (2022). Multifunctional structural composite supercapacitors based on MnO2-nanowhiskers decorated carbon fibers. J. Energy Storage.

[18] Artigas-Arnaudas J., Sánchez-Romate X.F., Sánchez M., Ureña A. (2023). Effect of electrode surface treatment on carbon fiber based structural supercapacitors: Electrochemical analysis, mechanical performance and proof-of-concept. J. Energy Storage.

[19] Cen T., Chen L., Zhang X., Tian Y., Fan X. (2021). A novel fiber-shaped asymmetric supercapacitor prepared by twisting carbon fiber/carbon nanotube/MnO_2_ and carbon fiber/carbon nanotube/polypyrrole electrodes+. Electrochim. Acta.

[20] Huang R., Zhang J., Dong Z., Lin H., Han S. (2022). Flexible carbon fiber/reduced-TiO_2_ composites for constructing remarkable performance supercapacitors. J. Power Sources.

[21] Saravanan R.S.A., Bejigo K.S., Kim S.-J. (2023). Scope and significance of transition metal oxide nanomaterials for next-generation Li-ion batteries. Mater. Chem. Front..

[22] Ayyanusamy P., Alphonse R., Minakshi M., Sivasubramanian R. (2024). Synthesis of amorphous nickel-cobalt hydroxides for Ni−Zn batteries. Chem. A Eur. J..

[23] Zhu J., Ding Y., Ma Z., Tang W., Chen X., Lu Y. (2022). Recent Progress on Nanostructured Transition Metal Oxides As Anode Materials for Lithium-Ion Batteries. J. Electron. Mater..

[24] Yadav S., Sharma A. (2021). Importance and challenges of hydrothermal technique for synthesis of transition metal oxides and composites as supercapacitor electrode materials. J. Energy Storage.

[25] Zhu X. (2022). Recent advances of transition metal oxides and chalcogenides in pseudo-capacitors and hybrid capacitors: A review of structures, synthetic strategies, and mechanism studies. J. Energy Storage.

[26] Haripriya M., Manimekala T., Dharmalingam G., Minakshi M., Sivasubramanian R. (2024). Asymmetric Supercapacitors Based on ZnCo_2_O_4_ Nanohexagons and Orange Peel Derived Activated Carbon Electrodes. Chem.–Asian J..

[27] Yuan C., Bin Wu H., Xie Y., Lou X.W. (2014). Mixed transition-metal oxides: Design, synthesis, and energy-related applications. Angew. Chem. Int. Ed..

[28] Li M., Meng Z., Feng R., Zhu K., Zhao F., Wang C., Wang J., Wang L., Chu P.K. (2021). Fabrication of bimetallic oxides (MCo_2_O_4_: M=Cu, Mn) on ordered microchannel electro-conductive plate for high-performance hybrid supercapacitors. Sustainability.

[29] Xu Y., Chu K., Li Z., Xu S., Yao G., Niu P., Zheng F. (2020). Porous CuO@C composite as high-performance anode materials for lithium-ion batteries. Dalton Trans..

[30] Zhang X., Du W., Lin Z., Tan X., Li Y., Ou G., Xu X., Lin X., Wu Y., Zeb A. (2022). Templated formation of Mn2O3 derived from metal-organic frameworks with different organic ligands as anode materials for enhanced lithium-ion storage. J. Alloy. Compd..

[31] Tan X., Wu Y., Lin X., Zeb A., Xu X., Luo Y., Liu J. (2020). Application of MOF-derived transition metal oxides and composites as anodes for lithium-ion batteries. Inorg. Chem. Front..

[32] Zhou J., Yang Q., Xie Q., Ou H., Lin X., Zeb A., Hu L., Wu Y., Ma G. (2022). Recent progress in Co–based metal–organic framework derivatives for advanced batteries. J. Mater. Sci. Technol..

[33] Wang Y., Li B., Zhang B., Tian S., Yang X., Ye H., Xia Z., Zheng G. (2020). Application of MOFs-derived mixed metal oxides in energy storage. J. Electroanal. Chem..

[34] Vanaraj R., Daniel S., Haldhar R., Asrafali S.P., Kim S.C. (2023). Direct growth of TiO_2_–MoO_2_/MnO_2_–MoO_2_ on plasma-treated carbon-cloth surface for high-performance supercapacitor and oxygen evolution reaction. Electrochim. Acta.

[35] Zhang S., Dai P., Liu H., Yan L., Song H., Liu D., Zhao X. (2021). Metal-organic framework derived porous flakes of cobalt chalcogenides (CoX, X = O, S, Se and Te) rooted in carbon fibers as flexible electrode materials for pseudocapacitive energy storage. Electrochim. Acta.

[36] Gong H., Bie S., Zhang J., Ke X., Wang X., Liang J., Wu N., Zhang Q., Luo C., Jia Y. (2022). In Situ Construction of ZIF-67-Derived Hybrid Tricobalt Tetraoxide@Carbon for Supercapacitor. Nanomaterials.

[37] Guan C., Zhao W., Hu Y., Lai Z., Li X., Sun S., Zhang H., Cheetham A.K., Wang J. (2017). Cobalt oxide and N-doped carbon nanosheets derived from a single two-dimensional metal–organic framework precursor and their application in flexible asymmetric supercapacitors. Nanoscale Horiz..

[38] Liu S., Kang L., Zhang J., Jung E., Lee S., Jun S.C. (2020). Structural engineering and surface modification of MOF-derived cobalt-based hybrid nanosheets for flexible solid-state supercapacitors. Energy Storage Mater..

[39] Dai S., Han F., Tang J., Tang W. (2019). MOF-derived Co3O4 nanosheets rich in oxygen vacancies for efficient all-solid-state symmetric supercapacitors. Electrochim. Acta.

[40] Lim G.J., Liu X., Guan C., Wang J. (2018). Co/Zn bimetallic oxides derived from metal organic frameworks for high performance electrochemical energy storage. Electrochim. Acta.

[41] Yang Q., Liu Y., Yan M., Lei Y., Shi W. (2019). MOF-derived hierarchical nanosheet arrays constructed by interconnected NiCo-alloy@NiCo-sulfide core-shell nanoparticles for high-performance asymmetric supercapacitors. Chem. Eng. J..

[42] Wang F., Han Q., Yi Z., Geng D., Li X., Wang Z., Wang L. (2017). Synthesis and performances of carbon fiber@Co3O4 based on metal organic frameworks as anode materials for structural lithium-ion battery. J. Electroanal. Chem..

[43] Han Q., Li X., Wang F., Han Z., Geng D., Zhang W., Li Y., Deng Y., Zhang J., Niu S. (2019). Carbon fiber@ pore-ZnO composite as anode materials for structural lithium-ion batteries. J. Electroanal. Chem..

[44] Huang T., Lou Z., Lu Y., Li R., Jiang Y., Shen G., Chen D. (2019). Metal-Organic-Framework-Derived MCo_2_O_4_ (M=Mn and Zn) Nanosheet Arrays on Carbon Cloth as Integrated Anodes for Energy Storage Applications. ChemElectroChem.

[45] Guan C., Liu X., Ren W., Li X., Cheng C., Wang J. (2017). Rational Design of Metal-Organic Framework Derived Hollow NiCo_2_O_4_ Arrays for Flexible Supercapacitor and Electrocatalysis. Adv. Energy Mater..

[46] Javed M.S., Aslam M.K., Asim S., Batool S., Idrees M., Hussain S., Shah S.S.A., Saleem M., Mai W., Hu C. (2020). High-performance flexible hybrid-supercapacitor enabled by pairing binder-free ultrathin Ni–Co–O nanosheets and metal-organic framework derived N-doped carbon nanosheets. Electrochim. Acta.

[47] Chen S., Wu J., Zhou R., Chen Y., Song Y., Wang L. (2015). Controllable growth of NiCo_2_O_4_ nanoarrays on carbon fiber cloth and its anodic performance for lithium-ion batteries. RSC Adv..

[48] Dai S., Yuan Y., Yu J., Tang J., Zhou J., Tang W. (2018). Metal–organic framework-templated synthesis of sulfur-doped core–sheath nanoarrays and nanoporous carbon for flexible all-solid-state asymmetric supercapacitors. Nanoscale.

[49] Fu Y., Zhou H., Hu Z., Yin S., Zhou L. (2020). Temperature-induced microstructure optimization of Co_3_O_4_ for the achievement of a high-areal-capacity carbon cloth-based lithium ion battery anode. Compos. Commun..

[50] Fang G., Zhou J., Liang C., Pan A., Zhang C., Tang Y., Tan X., Liu J., Liang S. (2016). MOFs nanosheets derived porous metal oxide-coated three-dimensional substrates for lithium-ion battery applications. Nano Energy.

[51] Liu T., Wang W., Yi M., Chen Q., Xu C., Cai D., Zhan H. (2018). Metal-organic framework derived porous ternary ZnCo_2_O_4_ nanoplate arrays grown on carbon cloth as binder-free electrodes for lithium-ion batteries. Chem. Eng. J..

[52] Dai Z., Long Z., Li R., Shi C., Qiao H., Wang K., Liu K. (2020). Metal–Organic Framework-Structured Porous ZnCo_2_O_4_/C Composite Nanofibers for High-Rate Lithium-Ion Batteries. ACS Appl. Energy Mater..

[53] Li H., Wang S., Feng M., Yang J., Zhang B. (2019). MOF-derived ZnCo_2_O_4_/C wrapped on carbon fiber as anode materials for structural lithium-ion batteries. Chin. Chem. Lett..

[54] Wu X., Meng L., Wang Q., Zhang W., Wang Y. (2019). Highly flexible and large areal/volumetric capacitances for asymmetric supercapacitor based on ZnCo_2_O_4_ nanorods arrays and polypyrrole on carbon cloth as binder-free electrodes. Mater. Lett..

[55] Feng M., Wang S., Yu Y., Feng Q., Yang J., Zhang B. (2017). Carboxyl functionalized carbon fibers with preserved tensile strength and electrochemical performance used as anodes of structural lithium-ion batteries. Appl. Surf. Sci..

[56] Gholampour N., Ahmadian-Yazdi M.-R. (2021). Investigation of zeolitic imidazolate frameworks–derived carbon nanotubes thin film in solar vapor generation. J. Porous Mater..

[57] Zhang Y., Chen Z., Tian J., Sun M., Yuan D., Zhang L. (2022). Nitrogen doped CuCo_2_O_4_ nanoparticles anchored on beaded-like carbon nanofibers as an efficient bifunctional oxygen catalyst toward zinc-air battery. J. Colloid Interface Sci..

[58] Mary A.J.C., Bose A.C. (2019). Incorporating Mn^2+^/Ni^2+^/Cu^2+^/Zn^2+^ in the Co_3_O_4_ Nanorod: To Investigate the Effect of Structural Modification in the Co_3_O_4_ Nanorod and Its Electrochemical Performance. ChemistrySelect.

[59] Kavinkumar T., Vinodgopal K., Neppolian B. (2020). Development of nanohybrids based on porous spinel MCo_2_O_4_ (M = Zn, Cu, Ni and Mn)/reduced graphene oxide/carbon nanotube as promising electrodes for high performance energy storage devices. Appl. Surf. Sci..

[60] Asghari A., Kazemi S.H., Khanmohammadi M. (2020). Facile and binder-free synthesis of N-doped carbon/ZnCo_2_O_4_ hybrid nanostructures on nickel foam for high-performance solid-state asymmetric supercapacitor. J. Mater. Sci. Mater. Electron..

[61] Xiao J., Yang S. (2011). Sequential crystallization of sea urchin-like bimetallic (Ni, Co) carbonate hydroxide and its morphology conserved conversion to porous NiCo_2_O_4_ spinel for pseudocapacitors. RSC Adv..

[62] Kamble G.P., Kashale A.A., Rasal A.S., Mane S.A., Chavan R.A., Chang J.-Y., Ling Y.-C., Kolekar S.S., Ghule A.V. (2021). Marigold micro-flower like NiCo_2_O_4_ grown on flexible stainless-steel mesh as an electrode for supercapacitors. RSC Adv..

[63] Luo Y., Zhang H., Guo D., Ma J., Li Q., Chen L., Wang T. (2014). Porous NiCo_2_O_4_-reduced graphene oxide (rGO) composite with superior capacitance retention for supercapacitors. Electrochim. Acta.

[64] Patil D.R., Koteswararao B., Begari K., Yogi A., Moussa M., Dubal D.P. (2019). Cobalt Cyclotetraphosphate (Co_2_P_4_O_12_): A New High-Performance Electrode Material for Supercapacitors. ACS Appl. Energy Mater..

[65] Wilson M.K., Saikrishna V., Mannayil J., Sreeja E.M., Abhilash A., Antony A., Jayaraj M.K., Jayalekshmi S. (2023). Exploring the potential of iron oxide nanoparticle embedded carbon nanotube/polyaniline composite as anode material for Li-ion cells. J. Mater. Sci. Mater. Electron..

