# Supplementary material for: M-Doped (M = Zn, Mn, Ni) Co-MOF-Derived Transition Metal Oxide Nanosheets on Carbon Fibers for Energy Storage Applications
Source: Nanomaterials (Basel). 2024 Nov 19;14(22):1846. doi: 10.3390/nano14221846 (PMC11597236; doi:10.3390/nano14221846)
Supplement: Supplementary file 1 [file nanomaterials-14-01846-s001.zip › nanomaterials-3328627-supplementary.pdf]

M-Doped (M = Zn, Mn, Ni) Co-MOF-Derived Transition Metal Oxide Nanosheets on Carbon Fibers for Energy Storage Applications

Andrés González-Banciella [1,*], David Martinez-Diaz [1], Adrián de Hita [1], María Sánchez [1,2,*] and Alejandro Ureña [1,2]

[1] Materials Science and Engineering Area, Escuela Superior de Ciencias Experimentales y Tecnología, Universidad Rey Juan Carlos, C/Tulipán s/n, 28933 Móstoles, Spain

[2] Instituto de Investigación de Tecnologías para la Sostenibilidad, Universidad Rey Juan Carlos, C/Tulipán s/n, 28933 Móstoles, Spain

* Correspondence: andres.banciella@urjc.es (A.G.-B.); maria.sanchez@urjc.es (M.S.)

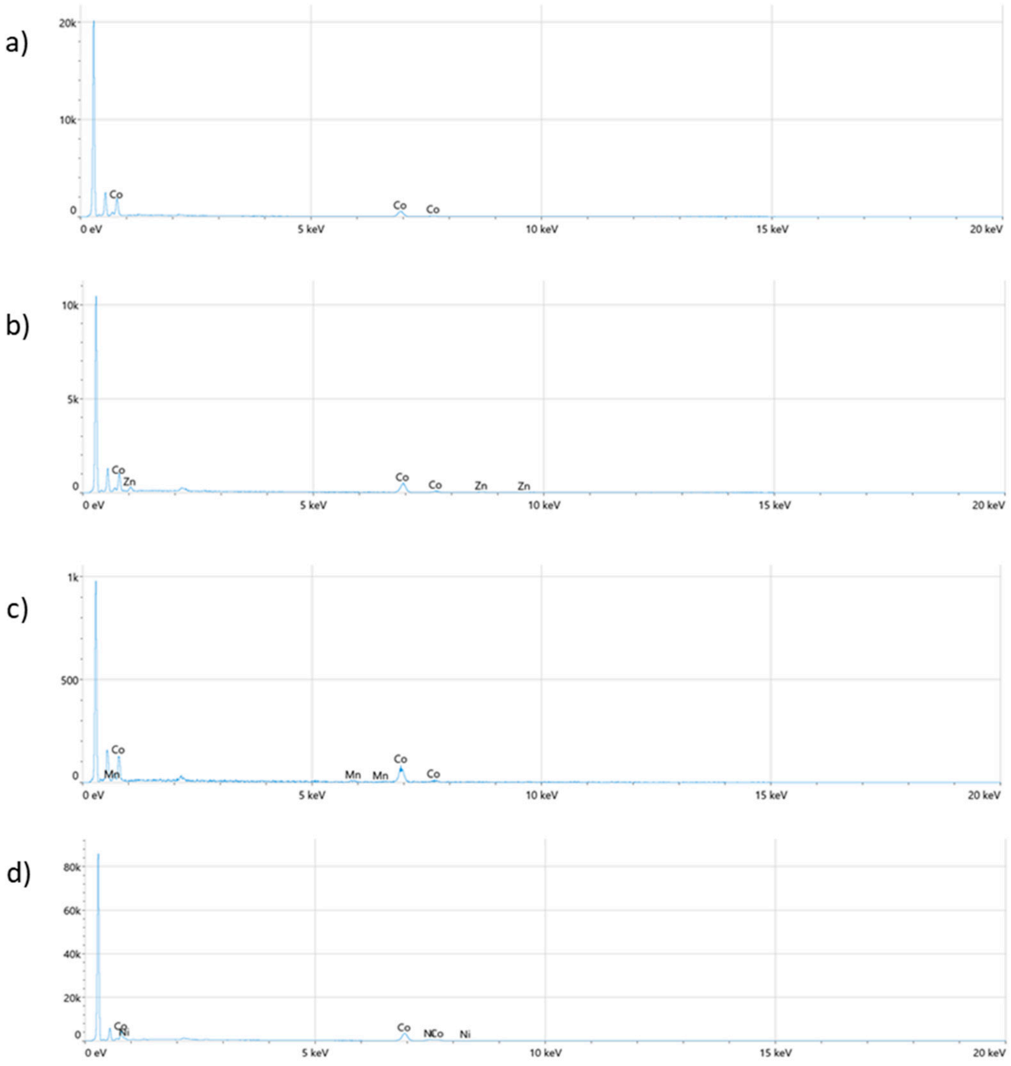

Figure S1: EDS spectra of no-doped sample (a), Zn-doped sample (b), Mn-doped sample (c) and Ni-doped sample (d).

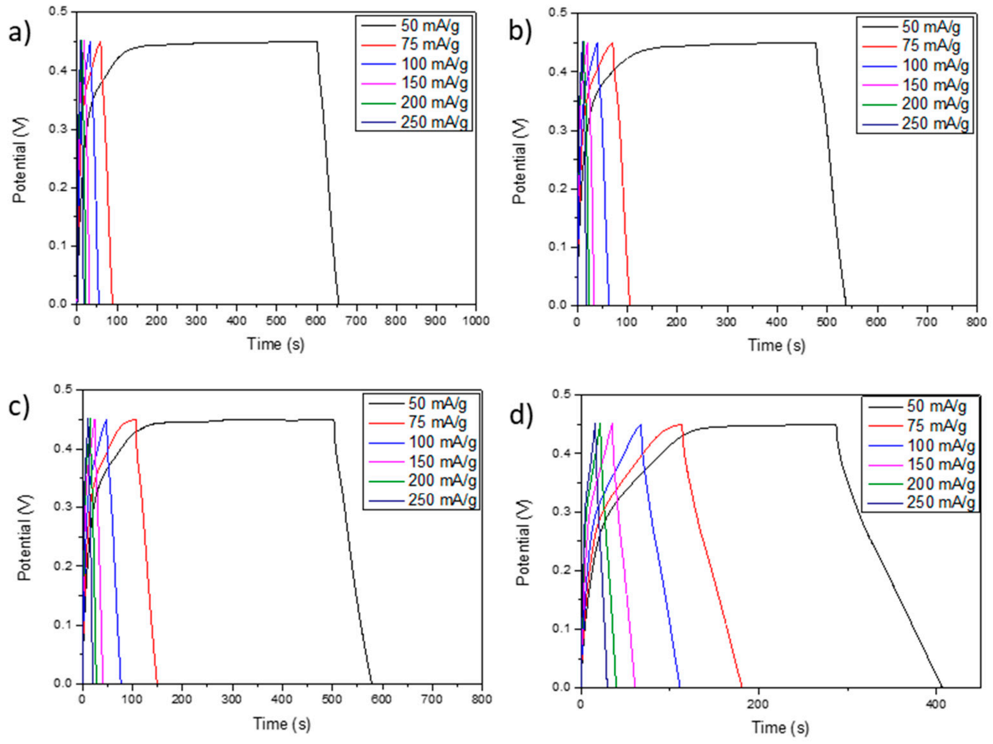

Figure S2: GCD test at different current densities of (a) undoped sample. (b) Zn-doped sample. (c) Mn-doped sample. (d) Ni-doped sample.

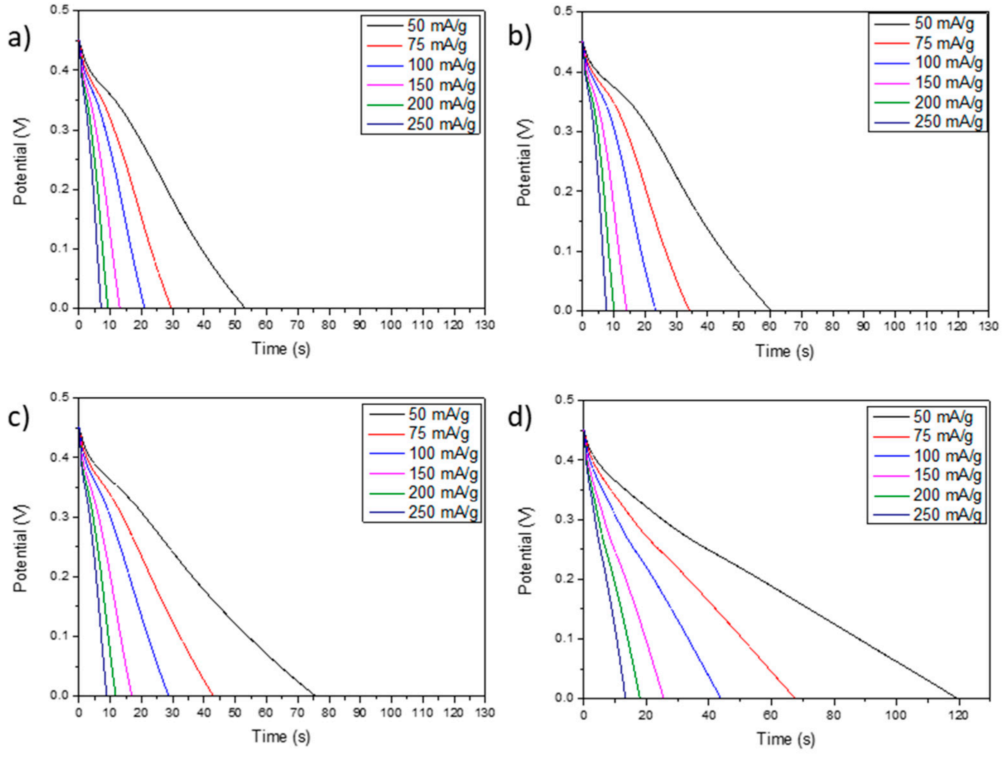

Figure S3: Galvanostatic discharges at different current densities of (a) undoped sample. (b) Zn-doped sample. (c) Mn-doped sample. (d) Ni-doped sample.
